# Study on the effect, safety, prognosis quality and application value of extracorporeal shock wave based neural activity in carpal tunnel syndrome patients

**DOI:** 10.1186/s12891-023-06285-1

**Published:** 2023-03-13

**Authors:** Haiou Zhang, Weiyan Zhao, Man Jiang, Yang Song

**Affiliations:** 1 Department of Hand and Foot Surgery, Affiliated Hospital of Beihua University, 132012 Jilin, China; 2 Department of orthopaedics, Affiliated Hospital of Beihua University, 132012 Jilin, China

**Keywords:** Extracorporeal shock wave, Nerve mobilization, Carpal tunnel syndrome, Pain score, Upper limb function

## Abstract

**Background:**

Mild to moderate CTS is the most common median nerve compression disease in middle-aged and elderly women, mainly manifested by hand numbness and pain. This paper analyzes the extracorporeal shock wave of patients with mild to moderate CTS after nerve mobilization.

**Methods:**

The clinical data of 92 patients with CTS from June 2020 to June 2022 are analyzed and randomly divided into extracorporeal shock wave group (n = 47) and routine group (n = 45). The routine group undergoes nerve mobilization, and the extracorporeal shock wave group receives extracorporeal shock wave therapy on the basis of the routine group. The clinical efficacy, symptom improvement, pain score, median nerve electrophysiological examination results, upper limb symptom and function scores, and ADL scores before and after treatment are observed. The *Spearman* correlation coefficient is used to analyze the correlation between upper limb function and ADL score, and the incidence of complications after treatment is analyzed.

**Results:**

The clinical efficacy, symptom improvement, pain score, median nerve electrophysiological examination results, upper limb symptom and function score, ADL score and the incidence of complications in the extracorporeal shock wave group are significantly better than those in the conventional group (*P* < 0.05). ADL scores are negatively correlated.

**Conclusion:**

Extracorporeal shock wave combined with nerve mobilization has a significant effect in the treatment of CTS patients, which can significantly improve the symptoms and pain scores of patients, and enhance the function of patients’ upper limbs. At the same time, the incidence of complications in patients is less, and it has high safety.

## Background

Mild-to-moderate CTS is a median nerve compression disease that occurs most frequently in middle-aged and elderly women, and is mainly manifested by hand numbness and pain [1, 2]. At present, the cause of CTS is still controversial. Analysis may be highly correlated with compression of the median nerve in the carpal tunnel. If not diagnosed and treated in time, it will seriously affect the patient’s upper limb and hand function, and reduce the patient’s quality of life [3, 4].

At present, conservative treatment is the main way to treat mild to moderate CTS, usually nerve mobilization, splint fixation, drug treatment, etc., which can relieve the symptoms of patients without surgery [5]. However, the efficacy of this method is poor and the effect is slow, and the clinical application value needs to be further improved. Therefore, in this study, extracorporeal shock wave combined with nerve mobilization is used to intervene in mild to moderate CTS, and to explore its clinical efficacy, safety and prognostic value in patients with mild to moderate CTS.

## Methods

### Baseline data

The baseline data of 92 CTS patients in our hospital from June 2020 to June 2022 were analyzed. The patient data were collected, sorted and divided into regular group and Extracorporeal shock wave group according to computer randomization method. Extracorporeal shock wave group (n = 47), male: 15 cases, female: 32 cases; aged 38–60 years, with an average age of (47.57 ± 3.69) years;12 patients were on the left side, 35 cases were on the right side; 25 cases were mild and 22 cases were moderate. Conventional group (n = 45), male: 16 cases, female: 29 cases; aged 39–62 years, mean age (46.57 ± 3.24) years old; 11 patients were on the left side, 34 cases were on the right; 26 cases were mild and 19 cases were moderate. The baseline data of the study subjects were not comparable (*P* > 0.05).

Inclusion criteria are as follows: (1) CTS diagnosed as mild to moderate [6]. (2) Patients with a course of disease less than 4 months. (3) Patients without mental illness and can communicate effectively. (4) First onset. Exclusion criteria are as follows: (1) Combined with other diseases affecting upper extremity function. (2) Insufficiency of important organs such as heart, lung, liver and kidney. (3) People with diabetes and other malignant diseases. (4) Unable to participate in the whole study.

### Treatment methods

The regular group was given routine intervention first, and during the treatment period, the patients were given methylcobalamin tablets produced by Eisai China Pharmaceutical Co., Ltd. The patient is instructed to protect the affected area at all times, and to fix the affected area in a neutral position with a splint when sleeping. At the same time, the patient was given nerve mobilization intervention, and the patient was instructed to lie in a supine position, with the head shifted to the healthy part, and the head and the shoulder of the affected part were close to the treatment bed. The doctor held the patient’s affected hand and supported the patient’s elbow with the thigh. The patient maintained the supination of the forearm and the back of the wrist. After that, the patient’s elbow and fingers were slowly stretched, and the patient’s shoulder was fully abducted after the patient’s elbow was completely straightened, and the patient’s shoulder was kept still for 10 s.

The Extracorporeal shock wave group was supplemented with extracorporeal shock wave therapy on the basis of the regular group. Instruct the patient to lie on his back, straighten the elbow and wrist of the affected side, palm up, the doctor stands on the affected side, fix the wrist joint of the affected side with one hand, hold the shock wave probe with the other hand, and hit the painful part of the affected part (probe voltage is 15 ~ 25kv, power energy density is 0.16mj/mm²). The total number of shocks in each treatment should not be less than 2000 times, 2 times per week, and 4 weeks as a cycle.

### Inspection method

All patients underwent electrophysiological examination of the median nerve according to Dantec Keypoint [7]. It mainly included thumb-wrist, middle finger-wrist SCV and SNAP. The SCV and SNAP amplitudes of the nerves in the thumb and middle finger were recorded according to the surface electrode, and 20% was added as the stimulation intensity on the basis of the maximum intensity of induced sensory nerves. DML and CMAP were recorded at the abdomen of the abductor brevis muscle based on surface electrodes.

### Observation indicators

(1) The clinical efficacy (excellent, good, moderate, poor) of the research subjects was analyzed. The excellent and good rate was the sum of excellent and good cases/total cases × 100%. The effective rate is the sum of excellent, good and moderate cases/total cases × 100% [8].

(2) Analyze the symptom improvement and pain score of the research subjects, and use the GSS to evaluate the patient’s symptom improvement [9]. VAS was used to evaluate the pain status of the research subjects. Score from 0 to 10, with 0 indicating no pain [10, 11].

(3) To explore the results of median nerve electrophysiological examinations before and after treatment, including DML, abductor pollicis brevis CMAP, thumb-wrist SCV, thumb-wrist SNAP, middle finger-wrist SCV, and middle finger-wrist SNAP.

(4) To explore the upper limb function and symptom scores of the research subjects, and to evaluate the patients’ wrist function and symptoms according to the BCTQ. The BCTQ symptom score includes 3 dimensions (numbness and pain degree, frequency and duration, as well as weakness and dysfunction), with a total of 11 items, each item is 1–5 points, a total of 11–55 points; The BCTQ function score is related to daily activities, with a total of 8 items (writing, holding a book, housework, buttoning buttons, carrying food bags, opening bottles, bathing and dressing, holding a telephone handle), each item is 1 ~ 5 points, and the total score is 8 ~ 40 points, the higher the score, the lower the daily activity ability [12];

(5) To analyze the daily activities of the research subjects, according to the ADL scale. The higher the score, the better the mobility [13].

(6) To explore the correlation between upper limb function and daily activities.

(7) The incidence of complications after treatment was analyzed.

### Statistical methods

Data was organized and entered into *SPSS 26.0* for data processing. Measurement data were expressed as mean ± standard deviation ($\overline x$ ± *s*), and *t*-test was used. The enumeration data were expressed as percentage (%), and the *x²* test was used. The comparison between groups at each time period was performed by repeated measures analysis of variance and spherical test. *Spearman* correlation coefficient was used to analyze the correlation between upper limb function and ADL score, P < 0.05, and the difference was statistically significant.

## Results

### Comparison of clinical efficacy of research subjects

Table 1 is the comparison of clinical efficacy of research subject. As shown in Table 1, the excellent rate and effective rate of the Extracorporeal shock wave group were higher than those of the regular group (*P* < 0.05).

**Table 1:** Comparison of clinical efficacy of research subjects(*n*,%)

group	number of examples	excellent	good	middle	difference	excellent rate	efficient
extracorporeal shock wave group	47	25(53.19)	20(42.55)	2(4.26)	0(0.00)	45(95.74)	47(100.00)
regular group	45	15(33.33)	19(42.22)	9(20.00)	2(4.45)	34(75.56)	43(95.56)
*x²*		9.574	0.362	8.557	9.241	8.774	9.532
*P*		<0.001	0.059	<0.001	<0.001	<0.001	<0.001

### Comparison of symptom improvement and pain score of research subjects

**Fig. 1:**
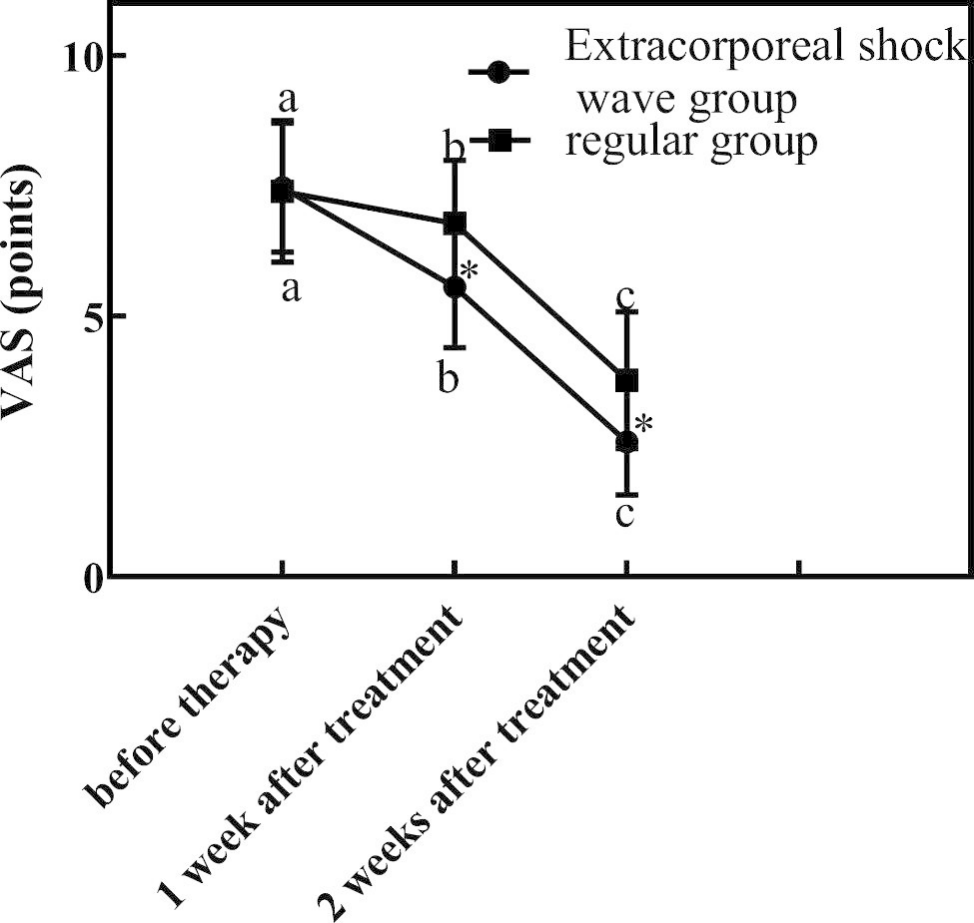
Comparison of VAS scores between the two groups before and after treatment

**Fig. 2:**
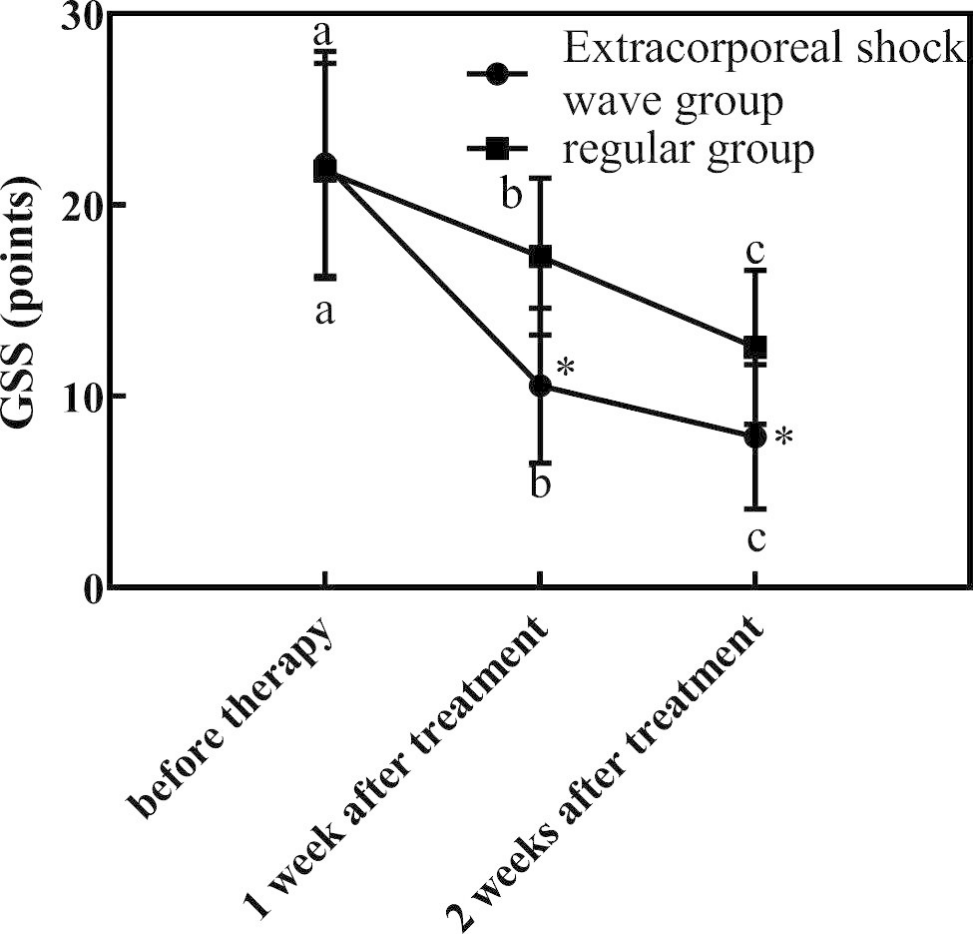
Comparison of GSS scores between the two groups before and after treatment

Table 2 is the comparison of symptom improvement and pain score of research subjects. Figure 1 is the comparison of VAS scores between the two groups before and after treatment. Figure 2 is the comparison of GSS scores between the two groups before and after treatment. Through the above experimental results, it can be observed that the VAS and GSS of the extracorporeal shock wave group were lower than those of the conventional group (P < 0.05) after treatment.

**Table 2:** Comparison of symptom improvement and pain score of research subjects($\overline x$ ± *s*)

group	time point	VAS (points)	GSS (points)
extracorporeal shock wave group (n = 47)	before therapy	7.47 ± 1.24	22.16 ± 5.87
	1 week after treatment	5.69 ± 1.16	14.16 ± 4.06
	2 weeks after treatment	3.26 ± 1.02	8.16 ± 3.79
regular group (n = 45)	before therapy	7.37 ± 1.36	22.12 ± 5.62
	1 week after treatment	6.23 ± 1.21	17.24 ± 4.11
	2 weeks after treatment	5.49 ± 1.31	11.24 ± 4.04
* F time*		8.264	9.278
*P time*		< 0.001	< 0.001
F time point*group		8.832	9.463
P time point*group		< 0.001	< 0.001

### Comparison of median nerve electrophysiological examination results before and after treatment

Table 3 is the comparison of median nerve electrophysiological examination results before and after treatment. “*” means compared with before treatment, *P < 0.05. “*#” means compared with extracorporeal shock wave group after treatment, *#P < 0.05. As shown in Table 3, the electrophysiological indexes of the extracorporeal shock wave group were better than those of the conventional group (P < 0.05) after treatment.

**Table 3:** Comparison of median nerve electrophysiological examination results before and after treatment($\overline x$ ± *s*)

group	time	DML(ms)	abductor pollicis brevis CMAP (mv)	thumb-wrist SCV (m/s)	thumb-Wrist SNAP (µv)	middle finger-wrist SCV (m/s)	middle finger-wrist SNAP (µv)
Extracorporeal shock wave group (n = 47)	before therapy	5.24 ± 1.34	4.38 ± 2.27	33.19 ± 7.65	8.50 ± 3.02	42.72 ± 7.62	9.45 ± 3.26
	After treatment	3.23 ± 0.59*	8.86 ± 3.17*	46.19 ± 5.48*	13.50 ± 4.17*	52.72 ± 7.89*	14.45 ± 3.62*
regular group (n = 45)	before therapy	5.17 ± 1.29	4.56 ± 2.19	33.32 ± 7.46	8.28 ± 3.18	43.00 ± 7.12	9.28 ± 3.74
	After treatment	3.62 ± 0.98*#	5.66 ± 3.01*#	41.20 ± 5.96*#	11.51 ± 4.09*#	48.81 ± 7.17*#	11.52 ± 3.49*#

### Comparison of upper extremity function and upper extremity symptom scores before and after treatment

Table 4 is the comparison of upper extremity function and upper extremity symptom scores before and after treatment. As shown in Table 4, the upper limb function and symptom scores of the subjects before treatment were not comparable (*P* > 0.05). The upper limb function and symptoms in the extracorporeal shock wave group were lower than those in the conventional group (*P* < 0.05).

**Table 4:** Comparison of upper extremity function and upper extremity symptom scores before and after treatment($\overline x$ ± *s*)

group	number of examples	BCTQ symptoms (points)		BCTQ function (points)	
		before therapy	after treatment	before therapy	after treatment
Extracorporeal shock wave group	47	35.40 ± 5.29	15.40 ± 2.78	28.76 ± 2.87	10.76 ± 2.04
regular group	45	34.81 ± 5.32	23.50 ± 2.89	28.26 ± 2.41	16.82 ± 2.15
*t*		0.741	9.676	0.659	9.228
*P*		0.089	<0.001	0.071	<0.001

### Comparison of the activities of living of the subjects before and after treatment

Table 5 is the comparison of the activities of daily living of the subjects before and after treatment. As shown in Table 5, the ADL scores of the research subjects before treatment were not comparable (*P* > 0.05). The activities of daily living in the extracorporeal shock wave group were better than those in the routine group (*P* < 0.05).

**Table 5:** Comparison of the activities of daily living of the subjects before and after treatment($\overline x$ ± *s*)

group	number of examples	ADL	
		before therapy	after treatment
Extracorporeal shock wave group	47	3.98 ± 2.55	7.98 ± 3.06
regular group	45	3.63 ± 2.73	6.48 ± 2.97
*t*		0.673	8.772
*P*		0.058	<0.001

### Analysis of the correlation between upper limb function and ADL score of research subjects

**Fig. 3:**
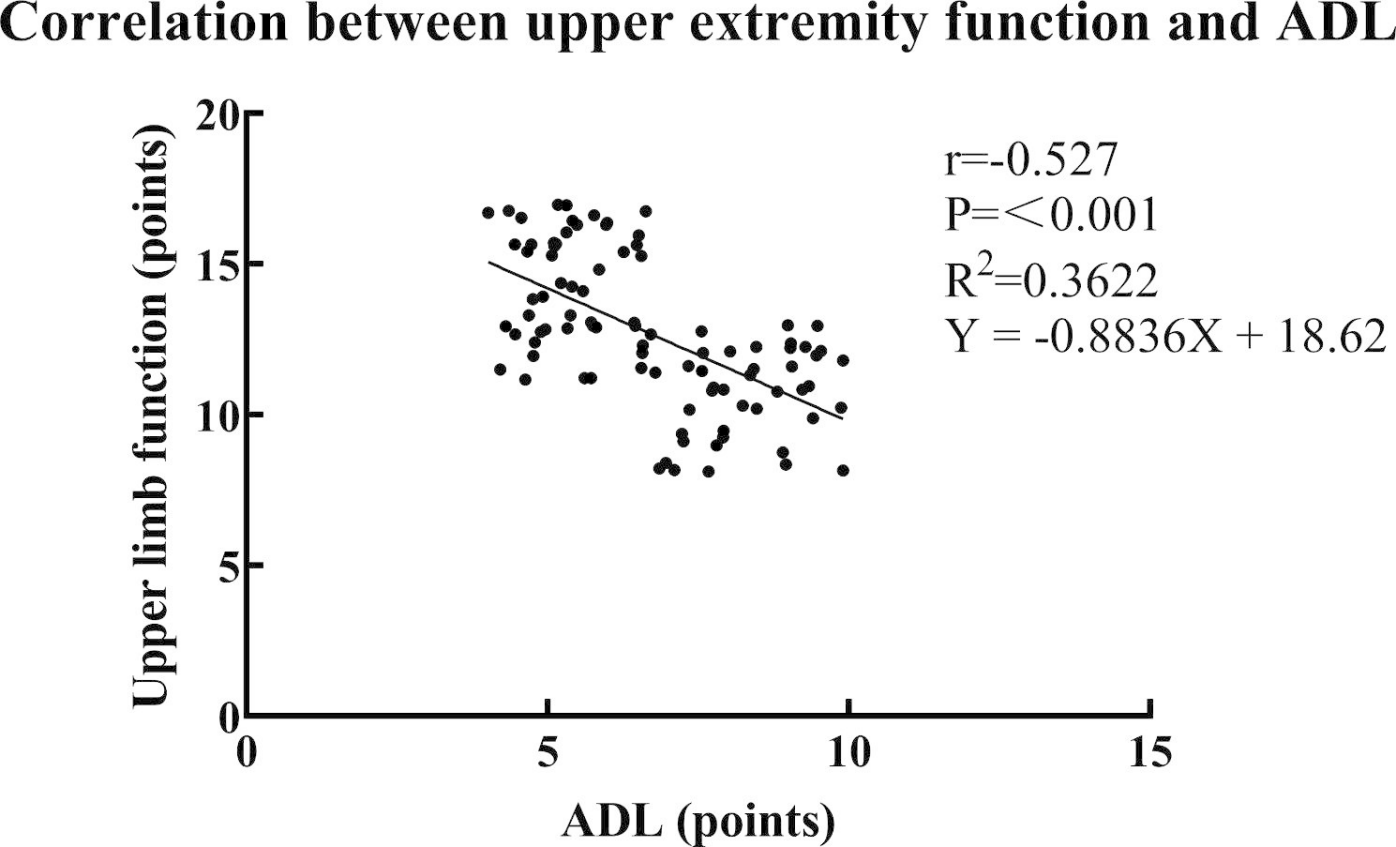
Correlation analysis of upper limb function and ADL score in patients

Table 6 is the correlation analysis of upper limb function and ADL score in patients. Figure 3 is the correlation analysis of upper limb function and ADL score in patients. Correlation analysis show that the upper limb function score was negatively correlated with the ADL score (*P* < 0.05).

**Table 6:** Correlation analysis of upper limb function and ADL score in patients

observation Indicator	ADL	
	*r*	*P*
upper limb function	-0.527	<0.001

### Comparison of the incidence of complications after treatment in the study subjects

Table 7 is the comparison of the incidence of complications after treatment in the study subjects. As shown in Table 7, the swelling of the hand, the numbness of the upper limbs and the decrease in the coordination of the wrist joint in the extracorporeal shock wave group were less than those in the conventional group (*P* < 0.05).

**Table 7:** Comparison of the incidence of complications after treatment in the study subjects(*n*,%)

group	Number of examples	swelling of the hand	upper limb numbness	decreased wrist coordination	total incidence
Extracorporeal shock wave group	47	2(4.26)	1(2.13)	1(2.13)	4(8.51)
regular group	45	6(13.33)	3(6.67)	3(6.67)	12(26.67)
*x²*		9.227	8.365	8.261	9.554
*P*		<0.001	<0.001	<0.001	<0.001

## Discussion

Nerve mobilization is a physiotherapy, mainly through the doctor’s bare-handed traction and expansion of the patient’s limb, thereby reducing the numbness and pain caused by the compression of the median nerve [14]. The extracorporeal shock wave is mainly conducted through the medium of physical mechanism, which is used to treat pain symptoms and promote tissue recovery. Relying on the positioning and movement of the therapeutic probe, it can effectively reduce the pain of patients [15, 16]. In this study, nerve mobilization combined with extracorporeal shock wave was used to treat CTS, and it was found that compared with simple nerve mobilization, it had a higher clinical effect, and the symptom improvement and pain score in the extracorporeal shock wave group were better than those in the conventional group. It showed that the two treatment methods had a certain synergistic effect. The reason was analyzed as follows: Nerve mobilization can reduce the pressure inside the nerve, thereby speeding up the blood circulation, further speeding up the patient’s nerve conduction, thereby relieving the patient’s pain. Extracorporeal shock waves mainly stimulate nerve endings, and the cavitation effect of shock waves can significantly eliminate local inflammation and relieve pain. Studies have shown that nerve mobilization has a positive effect on the results of median nerve electrophysiological examinations in CTS patients [17]. This paper shows that the extracorporeal shock wave group can significantly improve the electrophysiological indicators of the median nerve, suggesting that nerve mobilization combined with extracorporeal shock wave can significantly improve the recovery of damaged nerve function in patients with CTS. The mechanism is analyzed as follows: nerve mobilization can reduce nerve pressure, thereby reducing nerve adhesion, axoplasmic transport co-channel expansion, accelerating the inhibition of inflammatory mediator expression, reducing wrist nerve edema, and further improving neurophysiological sliding function.

This study also found that extracorporeal shock waves can improve upper extremity symptoms and function. The reason was analyzed as follows: the onset of CTS is mainly caused by excessive wrist strain or trauma, resulting in edema and adhesion of the nerve near the wrist. Nerve mobilization can shift the nerve during the treatment process, relieve the nerve adhesion, and stretch the affected wrist during the treatment process, which can scientifically and effectively exercise the affected area, speed up the recovery of the patient’s upper limb function, and relieve the symptoms of CTS. At the same time, extracorporeal shock waves can improve cell deformability, increase cell oxygen uptake, promote cell regeneration, stabilize the internal environment of the affected area, provide more nutritional support for nerve repair, and accelerate the recovery of upper limb function [18]. The extracorporeal shock wave group can improve the ADL score and the upper limb function of the patients is negatively correlated with ADL, indicating that the upper limb function was a protective factor for daily activities. Nerve mobilization combined with extracorporeal shock wave not only improves the clinical symptoms of patients, but also helps to continuously exercise the wrist and finger joints on the affected side, accelerates nerve closure, and enhances the patient’s grasping ability, thereby improving the patient’s upper limb function and further improving the patient’s self-care ability (wearing clothing, bathing, hand-held phone, etc.), to improve the activities of daily living of patients. Nerve mobilization can reduce the hypersensitivity of damaged nerves, reduce severe pain caused by small stimulation, restore nerve perception ability, thereby reducing upper limb numbness. At same time, extracorporeal shock waves have a certain inhibitory effect on inflammatory mediators, produce metabolic activation effects, and accelerate blood flow Circulation, reducing nerve swelling and adhesions, thereby relieving hand swelling. This paper also showed that extracorporeal shock waves can reduce the incidence of complications. It is suggested that nerve mobilization combined with extracorporeal shock wave in the treatment of CTS patients has high safety and can be widely used in clinical practice.

## Conclusion

Nerve mobilization combined with extracorporeal shock wave in the treatment of CTS patients has a good clinical effect, can significantly reduce the pain of patients, and has high safety. However, the selection range of the experimental objects in this study is narrow, and the results may not be used as an accurate basis. Therefore, it is necessary to further expand the scope of the research objects for deeper research to reduce errors.

## Data Availability

The datasets used and/or analyzed during the current study are available from the corresponding author on reasonable request.

## List of Abbreviations

CTS: Carpal Tunnel Syndrome
ADL: Activities of Daily Living
SCV: Sensory Conduction Velocity
SNAP: Sensory Nerve Active Potential
DML: Distal Motor Latency
CMAP: Compound Muscle Action Potential
GSS: Global Symptom Rating Scale
VAS: Visual analogue scale
BCTQ: Boston Carpal Tunnel Questionnaire
